# Orthotopic Heart Auto-Transplantation in a Swine Model

**DOI:** 10.4236/wjcs.2022.129017

**Published:** 2022-09

**Authors:** Michael P. Rogers, Gregory Fishberger, Nick Martini, Margaret Baldwin, Lei Wang, Wei Chen, Ruisheng Liu, Lucian Lozonschi

**Affiliations:** 1 Division of Cardiothoracic Surgery, Department of Surgery, University of South Florida Morsani College of Medicine, Tampa, FL, USA; 2 Department of Comparative Medicine, University of South Florida, Tampa, FL, USA; 3 Department of Molecular Pharmacology and Physiology, University of South Florida Morsani College of Medicine, Tampa, FL, USA; 4 Department of Physics, College of Arts and Sciences, University of South Florida, Tampa, FL, USA

**Keywords:** Porcine, Cardiac Surgery, Cardiac Transplantation

## Abstract

Background and Aim: The porcine heart bears the best resemblance to the human heart and remains the preferred preclinical model for anatomical, physiological, and medical device studies. In an effort to study phenomena related strictly to ischemia reperfusion and donor preservation protocols, it is essential to avoid the immune responses related to allotransplantation. Orthotopic auto-transplantation is a unique strategy to the field of cardiac transplantation for *ex vivo* experimentation. Nevertheless, auto-transplantation carries its own technical challenges related to insufficient length of the great vessels that are to be transected and re-anastomosed. Methods: A novel method for orthotopic cardiac auto-transplantation in the porcine model was developed and was described herein. Porcine models were used for *ex vivo* experimentation of a novel device to study ischemia reperfusion injury. Results: A total of five porcine models were used for *ex vivo* experimentation of a novel device to mitigate ischemia reperfusion injury and determine effects of donor preservation. Modifications to routine cardiac transplantation protocols to allow for successful auto-transplantation are described. Conclusion: Orthotopic cardiac auto-transplantation in the porcine model is a plausible and technically feasible method for reliable study of ischemia reperfusion injury and donor preservation protocols. Here, we describe methods for both direct orthotopic porcine cardiac auto-transplantations as well as a simplified protocol that can be substituted for full surgical auto-transplantation for the studies of preservation of donor hearts.

## 1. Introduction

Pigs and humans have anatomic and physiologic similarities. By comparison, humans share far more immune-related genes and proteins with pigs than they do with mice or other animals [1] [2] [3]. Additionally, swine models appear to develop similar clinical and histopathological features of primary graft dysfunction to that seen in humans after transplantation [4]. The porcine heart is often considered the most anatomically similar to the human heart when using large animals. As such, it is an ideal platform to perform cardiac surgical research. Growing interest in the feasibility of using genetically modified porcine hearts for porcine-to-human cardiac xenotransplantation highlights this relevance [5] [6] [7]. In this work, we present our direct approach to orthotopic porcine cardiac auto-transplantation as well as a simplified protocol that can be substituted for full surgical auto-transplantation for conducting research in the preservation of donor hearts.

## 2. Protocol

For the purposes of our study, adult Yorkshire pigs (60 – 80 kg) were used for heart size similarity to an adult human. The animal was procured from a local Institutional Animal Care and Use Committee (IACUC) approved vendor. This study received local Institutional Review Board (IRB) approval and strictly conformed to the institutional IACUC standards, as well as the “Guide for the Care and Use of Laboratory Animals” prepared by the Institute of Laboratory Animal Resources [8].

### 2.1. Donor Anesthesia Induction and Procedural Preparation

The animal was initially premedicated with ketamine (20 mg/kg), atropine (0.04 mg/kg), and diazepam (2 mg/kg), followed by anesthetic induction and maintenance with inhalational isoflurane. Isoflurane was titrated for an end tidal concentration of 1% - 3%, and 3 L/min 100% FiO_2_ were delivered by face mask. To ensure adequate anesthesia dosing, jaw tone and the absence of pain during toe pinch were assessed. Orotracheal intubation followed using a size 7.5 – 8.5 mm endotracheal tube. To assess oxygen saturation, continuous monitoring was determined by probes placed on the ear or bottom lip and serial arterial blood gas measurements were collected throughout the operation (Table 1).

Peripheral intravenous access was obtained by 20-gauge Angiocath insertion in the ear vein with an initial maintenance infusion of 0.9% NaCl. Using the modified-Seldinger technique, a percutaneous central venous sheath introducer was placed in the right jugular vein after placement of the animal in Trendelenburg position. An arterial line for arterial blood pressure monitoring was placed in the left common carotid artery for blood pressure monitoring.

### 2.2. Peripheral Cannulation for Cardiopulmonary Bypass

**Figure 1.**
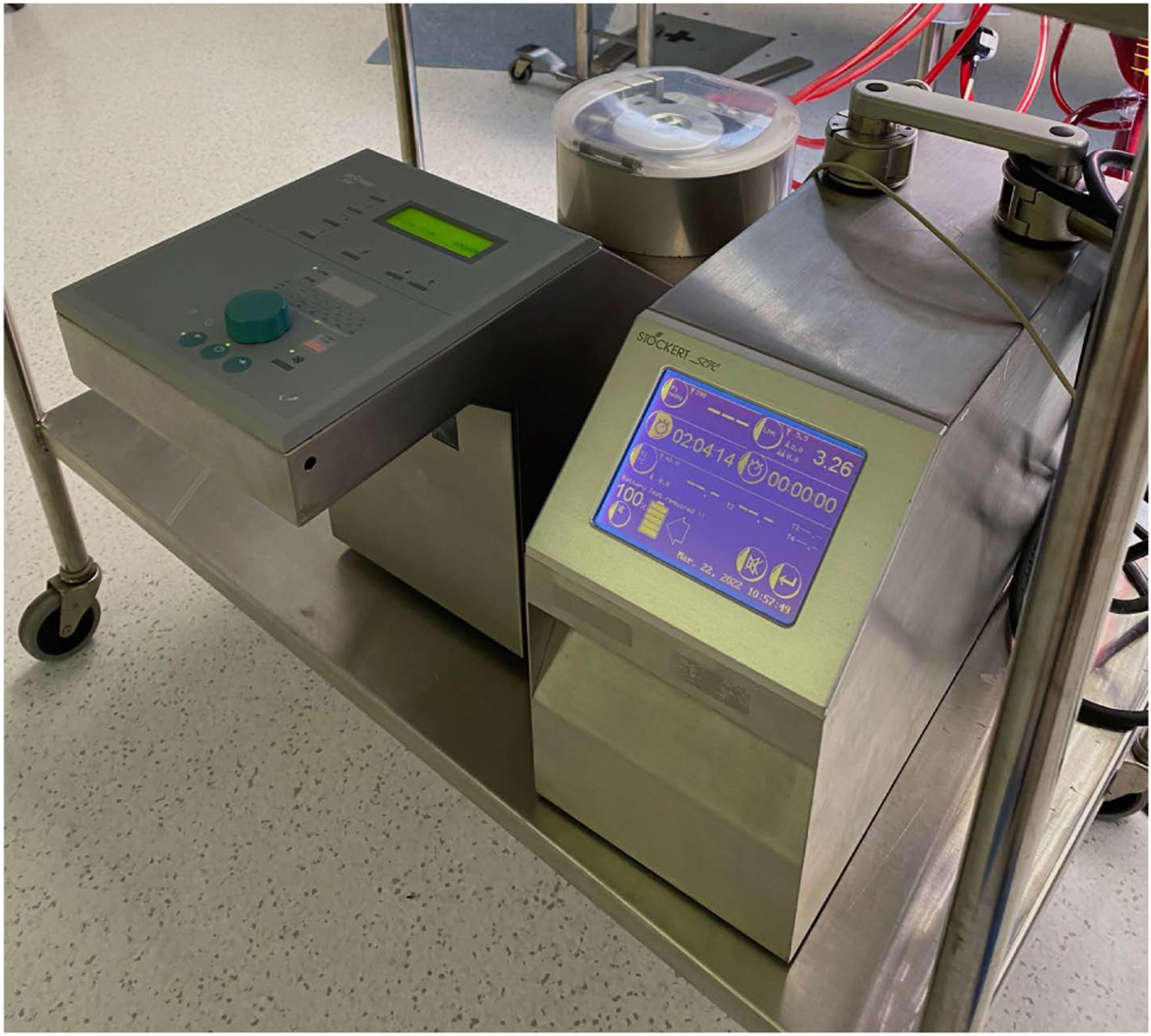
Modified cardiopulmonary bypass circuit consisting of a Sorin/Stöckert SCP centrifugal pump (LivaNova PLC, London, United Kingdom) and Medtronic (Medtronic Inc., Minneapolis, MN) tubing and reservoir.

The left and right inguinal areas were prepped and draped in the usual sterile fashion. The left common femoral vein and right common femoral artery were accessed percutaneously and a guide wire advanced into the inferior vena cava and descending aorta, respectively. This step was performed after sternotomy was completed to allow for manual confirmation of the wire position. A full dose of 300 IU/kg of heparin was given to achieve a target activated coagulation time (ACT) of 400 seconds or greater. A standard approach for percutaneous femoral cannulation using the modified-Seldinger technique was used to serially dilate the femoral vessels and place the femoral cannulas. A 15 Fr. femoral arterial and 17 Fr. venous cannula (Medtronic Inc., Minneapolis, MN) were used. Cannulas were connected to a modified cardiopulmonary bypass circuit consisting of a Sorin/Stöckert SCP centrifugal pump (LivaNova PLC, London, United Kingdom) and Medtronic (Medtronic Inc., Minneapolis, MN) tubing and reservoir (Figure 1). The cardiopulmonary bypass (CPB) circuit was primed with 700 cc of crystalloid solution (e.g., Plasmalyte or normal saline), with 25 grams of mannitol, 10,000 units of Heparin, and 50 mill equivalents of sodium bicarbonate.

### 2.3. Donor Heart Procurement

**Figure 2.**
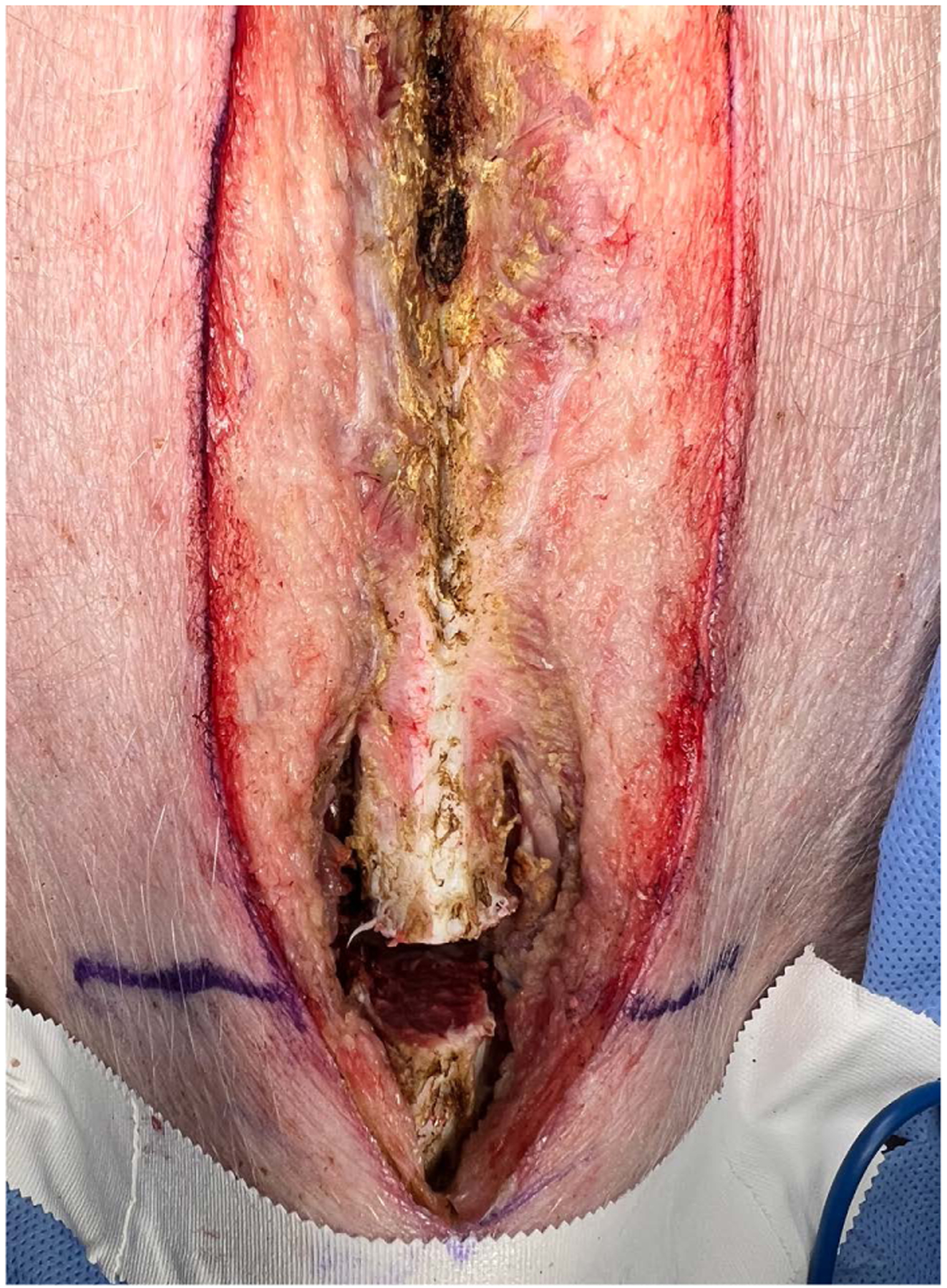
Preservation of the manubrium was considered for locomotion of the animal in future survival studies. ALebsche knife was used to divide the sternum from the manubrium at the level of the 2nd intercostal space.

A mid-line sternotomy incision was performed with a #10 blade followed by electrocautery from the mid-cervical region to below the xiphoid process. An area approximately three finger breadths below the manubrium was chosen as the superior margin of the sternotomy. Preservation of the manubrium was considered for locomotion of the animal in future survival studies. Using a Lebsche knife (Novo Surgical, Westmont, IL), the sternum was separated from the manubrium at the level of the 2nd intercostal space (Figure 2). The remainder of the sternum was then divided using a standard sternal saw from the xiphoid process to the superior margin in a “T” fashion. Hemostasis was carefully achieved, two laparotomy sponges were placed on either side of the sternum, and a sternal retractor was placed. The thymus was then dissected and excised. This was followed by incision of the pericardium with placement of pericardial stay sutures. Using electrocautery, the aortopulmonary space was dissected and the anterior aspect of the ascending aorta and arch was dissected to the level of the left subclavian artery. To isolate and secure the superior vena cava (SVC) and inferior vena cava (IVC), spaces between these vessels, the innominate artery, and the pericardium were dissected with encircling of the SVC and IVC by umbilical tapes. The SVC was cannulated for cardiopulmonary bypass using a 4–0 prolene purse-string suture and a 20 Fr. (Edwards Lifesciences, Irvine, CA) right angle single-stage venous cannula (e.g., DLP single lumen angled venous cannula) and connected to the inferior femoral venous cannula in a Y-configuration. Cannulas were connected to the bypass circuit using a “3/8-3/8-1/2” Y-connector with adequate deairing. CPB was initiated with a target cardiac index of >2.2 – 2.5 L/min/m^2^ while maintaining mean arterial pressure > 55 – 75 mmHg. Targeted mild hypothermia to 34 degrees centigrade was initiated. Arterial blood gases were monitored every 45 minutes and ACTs monitored every 30 minutes to ensure ACT > 450 seconds. A 12-gauge antegrade cardioplegia catheter was placed in the proximal ascending aorta.

Standard preparation del Nidocardioplegia solution was prepared and connected to the antegrade delivery cannula followed by aortic cross-clamping on the distal ascending aorta. A full dose of 1.5 L of cardioplegia solution at 4°C was given with a target root pressure of 80 – 100 mmHg. To achieve organ cooling, saline ice slush was placed in the pericardial cavity and over the heart. After cardioplegia infusion, cardiectomy was performed in the usual cardiac transplant fashion, following by placement of the heart into an organ bag with 500 mL of preservation (del Nidocardioplegia) solution. Of note, the porcine model always has a significant left azygous vein present (comparable to the much-rarer left superior caval or oblique vein in humans) that enters the coronary sinus [9]. This must be ligated to allow for bloodless cardiectomy. Additionally, the porcine left atrium receives only two pulmonary veins, with the superior and inferior pulmonary vein confluence occurring prior to reaching the atrium. This results in two pulmonary veins entering the left atrium close to each other, as opposed to left and right superior and inferior pulmonary venous drainage in humans. Particular attention to dissection in this area to allow for sufficient left atrial cuff for anastomosis is critical, as the left and right pulmonary veins are quite diminutive and often not sufficient for primary anastomosis. It is essential to perform a meticulous dissection during cardiectomy as this can aid in obtaining additional length for the SVC, IVC, and pulmonary artery.

The *ex vivo* experiments occurred for a total of two hours during which time the porcine model was maintained on cardiopulmonary bypass. Two cardiopulmonary bypass sumps were placed in the pericardial cavity as well as the pulmonary veins to maintain return of all blood and adequate pump volume.

### 2.4. Auto-Reimplantation Procedure

The heart was prepared for re-implantation. Biatrial anastomotic technique was followed in the order of left atrium, IVC, pulmonary artery, aorta and SVC. The anastomosis of divided cuff of each great vessel was challenging due to minimal redundancy. In two animals, we first anastomosed extension cuffs consisting of custom-fashioned Dacron (Vascutek^®^ Gelseal^™^, Terumo Cardiovascular Systems Co., Ann Arbor, MI, USA) grafts while the heart remained in its preservation basinto complete the IVC and left atrial anastomoses due to distance mismatch. Following all anastomoses, the aortic cross clamp was then removed, and hemostasis was ensured.

The donor heart was reperfused on CPB as needed while ventricular arrythmia was treated with internal defibrillation, if required. Ventricular pacing was utilized in all cases and antiarrhythmic medications (e.g., amiodarone, lidocaine, and magnesium sulfate) were administered. Cardiopulmonary bypass was then weaned in a routine fashion. The central venous line was used to monitor central venous pressure with a target of 10 – 15 mmHg. As required, vasoactive and inotrope continuous infusions (e.g., dobutamine, epinephrine, vasopressin, and milrinone) were initiated. Volume replacement was returned from CPB as needed, and heparin reversal was achieved with protamine.

## 3. Discussion

Orthotopic auto-transplantation is a unique strategy to the field of cardiac transplantation for *ex vivo* experimentation. This approach eliminates the need to match the immunological profiles of donor and recipient, thus preventing acute immunologic organ rejection and precludes the need for immunosuppression medication. Our protocol demonstrates the feasibility of this strategy for cardiovascular translational research. The main barrier with this method is inadequate length on both donor and receiving cuffs of the great vessels for creating of tension free anastomoses. This limitation can be overcome with the anastomosis of polyester cuff extenders matched in size and diameter to the great vessels’ cuffs of the explanted heart before re-transplantation.

**Figure 3.**
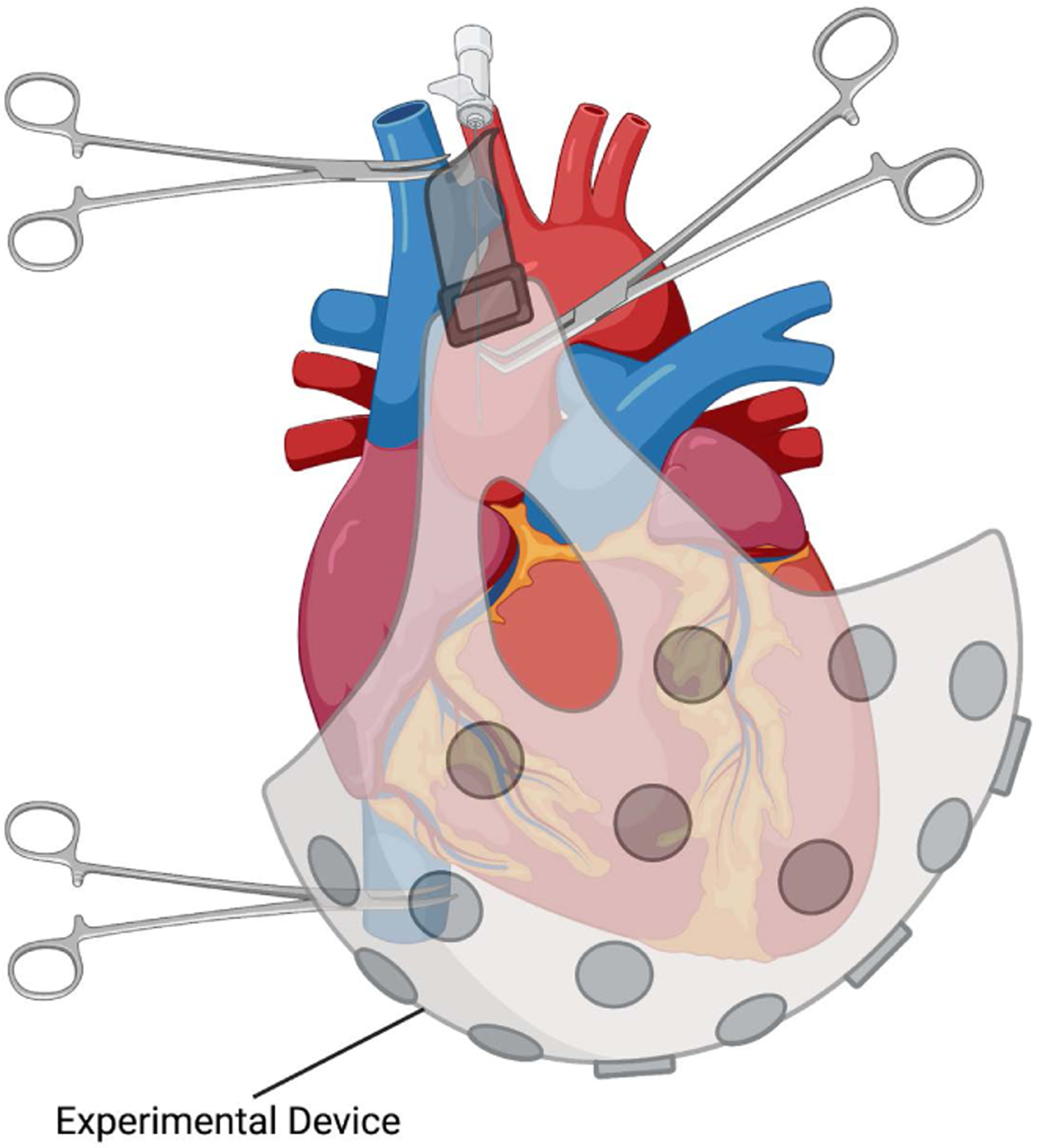
A proposed method for *in situ* cardiac circulatory isolation under cardioplegic arrest.

An alternative to the method described above is by altogether avoiding complete cardiectomy for device testing through *in situ* cardiac circulatory exclusion and cardioplegic arrest. The aorta is cross-clamped and antegrade cardioplegia is delivered in the usual fashion to establish full arrest, after the SVC/IVC inflows are occluded (Figure 3). Both left and right atria are vented through small atriotomies to completely empty the heart. The heart is thus excluded from circulation and maintained at 4 degrees Celsius with a specially designed cooling device. This technique obviates the need for great vessels’ division and re-anastomosis and the possible complications thereof (*i.e*., kinking, size mismatch, need for Dacron grafts extension for creation of tension free anastomosis). Once the designed experiment has been completed, the heart is reperfused in the normal fashion as described above.

## Figures and Tables

**Table 1.** Variables monitored during experimentation and during cardiopulmonary bypass.

Monitored Variables
Blood Pressure with Arterial Line
Pulse Rate
Oxygen Saturation
Central Venous Pressure
Activated Coagulation Time
Basic Metabolic Panel
Hemoglobin and Hematocrit
Arterial Blood Gas
Cardiac Index
